# Searching for bidirectional promoters in *Arabidopsis thaliana*

**DOI:** 10.1186/1471-2105-10-S1-S29

**Published:** 2009-01-30

**Authors:** Quan Wang, Lin Wan, Dayong Li, Lihuang Zhu, Minping Qian, Minghua Deng

**Affiliations:** 1 Center for Theoretical Biology, Peking University, Beijing100871, PR China; 2 LMAM, School of Mathematical Sciences, Peking University, Beijing 100871, PR China; 3 State Key Laboratory of Plant Genomics, Institute of Genetics and Developmental Biology, Chinese Academy of Sciences, Beijing 100101, PR China

## Abstract

**Background:**

A "bidirectional gene pair" is defined as two adjacent genes which are located on opposite strands of DNA with transcription start sites (TSSs) not more than 1000 base pairs apart and the intergenic region between two TSSs is commonly designated as a putative "bidirectional promoter". Individual examples of bidirectional gene pairs have been reported for years, as well as a few genome-wide analyses have been studied in mammalian and human genomes. However, no genome-wide analysis of bidirectional genes for plants has been done. Furthermore, the exact mechanism of this gene organization is still less understood.

**Results:**

We conducted comprehensive analysis of bidirectional gene pairs through the whole *Arabidopsis thaliana *genome and identified 2471 bidirectional gene pairs. The analysis shows that bidirectional genes are often coexpressed and tend to be involved in the same biological function. Furthermore, bidirectional gene pairs associated with similar functions seem to have stronger expression correlation. We pay more attention to the regulatory analysis on the intergenic regions between bidirectional genes. Using a hierarchical stochastic language model (HSL) (which is developed by ourselves), we can identify intergenic regions enriched of regulatory elements which are essential for the initiation of transcription. Finally, we picked 27 functionally associated bidirectional gene pairs with their intergenic regions enriched of regulatory elements and hypothesized them to be regulated by bidirectional promoters, some of which have the same orthologs in ancient organisms. More than half of these bidirectional gene pairs are further supported by sharing similar functional categories as these of handful experimental verified bidirectional genes.

**Conclusion:**

Bidirectional gene pairs are concluded also prevalent in plant genome. Promoter analyses of the intergenic regions between bidirectional genes could be a new way to study the bidirectional gene structure, which may provide a important clue for further analysis. Such a method could be applied to other genomes.

## Background

**Figure 1:**
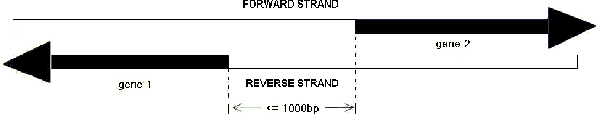
**A sketch map of bidirectional promoter**. Gene 1 and gene 2 are from the same chromosome, and gene 1 is on the reverse stand while gene 2 is on the forward one. The distance between their transcription start sites (TSSs) is not more than 1000 base pairs. Gene 1 and gene 2 are called "bidirectional genes" and "bidirectional promoter" refers to the intergenic region separating "bidirectional genes" generally.

With the development of sequencing techniques, more and more genomes are available now, making the genome-scale sequence studies possible, such as the gene structure prediction, gene organization identification and so on. In genome sequence, there is one kind of gene pairs which are arranged head-to-head on opposite strands and with no more than 1000 base pairs separating their transcription start sites (TSSs) [1,2]. Such an arrangement is previously defined as "bidirectional" and the divergent gene pairs are termed as "bidirectional genes", while the intergenic region between a "bidirectional gene pair" is often called a "bidirectional promoter" (Figure 1 – A sketch map of bidirectional promoter.). Individual examples of bidirectional genes as well as a few genome-wide analyses have been known in mammalian and human genomes for years [1-8]. The "head-to-head" gene organization was first observed in the mouse DHFR gene [3]. Another example was found by Gavalas et al in the analysis of the chicken GPAT/AIRC bidirectional promoter [4]. Adachi et al discovered that bidirectional gene organization is a common architectural feature of the human genome and Trinklein et al have made a systematic analysis of bidirectional genes in human genome, finding that they are more correlated in expression and often function in DNA repair [1,2]. Li et al analyzed the bidirectional genes using human, chicken and *fugu *genomic data and concluded that this gene organization is ancient and conserved, which subjects functionally related genes to correlated transcriptional regulation and thus provides an exquisite mechanism of transcriptional regulation based on gene organization [7]. To our knowledge, no genome-wide analysis of bidirectional genes for plant genome has been reported until now.

In our study, we pay more attention to the regulatory analysis of the intergenic region between bidirectional genes. In the papers mentioned above, a "bidirectional promoter" is presumedly defined as the intergenic DNA sequence between two TSSs of a bidirectional gene pair. However, increasing evidences show that in eukaryotic organisms, promoters are not always in the vicinity of TSSs of genes, but can locate from the distant 5' upstream regions of genes, or to the 3' downstream regions [9,10]. Recently the ENCODE pilot project even suggested that the human genome is pervasively transcribed [11] and it is noted that most genes are controlled cooperatively by several transcription factors (TFs) binding to various regulatory elements [12,13] which are often located spatially close to each other. Therefore, in the scrutiny of intergenic DNA sequences between bidirectional genes, an additional criterion was used here to see whether those sequences are also enriched of regulatory elements which are essential for the initiation of transcription. The hierarchical stochastic language (HSL) model we recently developed [14] were utilized to identify putative transcriptional regulatory regions (TRRs) on those intergenic sequences between bidirectional genes by scoring them. The TRR regions identified by HSL are enriched of cooperative motif pairs which are over-represented in promoters. We found that these cooperative motif pairs selected from yeast are conserved across species and HSL trained on yeast are successfully applied to identify promoters (functional regions) for fly, human, *Arabidopsis thaliana*, and rice [14].

In this article, we conducted a genome-wide survey of *Arabidopsis thaliana *genome and identified 2471 bidirectional gene pairs in the whole genome (excluding chloroplast genome and mitochondrial genome). Besides examining the expression and function association of bidirectional genes as previous studies [2,7], we searched the intergenic DNA sequences between bidirectional genes by HSL to see if they contain TRR. We found that these 2471 bidirectional gene pairs show a higher probability to be coordinately regulated and functionally associated which are consistent with results in mammalian [2]; and thus indicate that this "head-to-head" gene organization is prevalent in *A. thaliana *genome. We further showed that functionally associated bidirectional genes have higher frequencies to contain intergenic sequences with higher TRR scores, indicating that these sequences response for co-regulating the bidirectional genes. Interestingly, most of the intergenic DNA sequences of bidirectional genes with high TRR scores contain only one peak of TRR score, suggesting that the gene pair share the same promoter. Finally, we picked 27 functionally associated bidirectional gene pairs with high TRR scores and hypothesized them to be regulated by bidirectional promoters. Five of them share common ancient orthologs and in addition, more than half of them are further supported by sharing the same functional categories (GO terms) as these of handful experimental verified bidirectional genes in other organisms.

## Results

### Identify bidirectional genes

**Figure 2:**
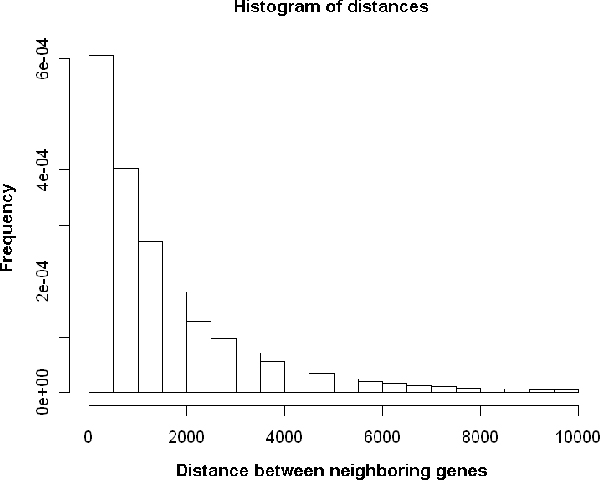
Histogram of distances between neighboring genes of *A. thaliana*.

We calculated distances between the TSSs of neighboring gene pairs among the whole *Arabidopsis thaliana *genome, excluding chloroplast genome and mitochondrial genome (Figure 2 – Histogram of distances between neighboring genes of *A. thaliana*). 14860 pairs have a separating region not more than 1000 bp, while only 2471 pairs are arranged "head-to-head" on opposite strands, accounting for approximately 13.3% of all the 37019 *A. thaliana *genes (See Methods for details). Of the 2471 bidirectional gene pairs, the max distance between TSSs is 1000 base pairs while the minimum is -932 base pairs (minus distances mean overlapping transcripts). There are 67 pairs with distances less than 0, meaning 2.7% pairs have genes whose transcripts are predicted to overlap at the 5' ends, whereas 2404 pairs (97.3%) are nonoverlaping. Interestingly, the majority (82%) of the 2404 nonoverlaping bidirectional promoters are more than 200 base pairs in length. For comparison, we randomly chose 10000 gene pairs to give a contrast of bidirectional genes, which will be served as background references through the following analysis.

### Coexpression of bidirectional genes

**Figure 3:**
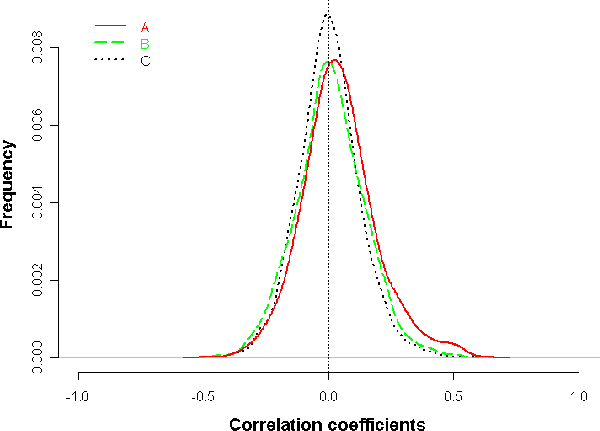
**Distribution of Pearson correlation coefficients**. (A) The distribution of correlation coefficients of bidirectional genes average over 46 microarray data sets. (B) The distribution of correlation coefficients of neighboring genes average over 46 microarray data sets. (C) The distribution of correlation coefficients of randomly paired genes average over 46 microarray data sets. Bidirectional gene pairs are more correlated in expression than randomly paired genes (P < 2.2 × 10^-16 ^by the Wilcoxon rank sum test) and neighboring gene pairs (P = 1.17 × 10^-11^).

For a potential of sharing common *cis*-regulatory elements, the two genes from one bidirectional pair tend to be coordinately regulated than randomly paired genes in expression level [15,16]. We calculated the Pearson correlation coefficients of expression for bidirectional gene pairs as well as the randomly picked gene pairs over 46 data sets independently, where microarray data are derived from AtGenExpression project (See Methods for details). For 1392 bidirectional gene pairs having complete gene expression data available, their averaged correlation coefficients (over 46 data sets) are significantly correlated than that of random pairs (Figure 3 – Distribution of Pearson correlation coefficients). Previous studies showed that genes in a neighborhood region are often coexpressed [17,18], so we also computed the Pearson correlation coefficients of neighboring genes in *Arabidopsis thaliana *genome (See Methods for details of identification of neighboring genes). Figure 3 – Distribution of Pearson correlation coefficients. shows that bidirectional genes are more correlated in expression than random gene pairs (P < 2.2 × 10^-16^) and neighboring genes (P = 1.17 × 10^-11^) by the Wilcoxon rank sum test. Setting the 95^th ^percentiles of the random pairs' correlation distribution as a cutoff of coordinately regulation, we find 170 pairs (12.21%) of the 1392 pairs with correlation coefficients are more correlated in expression. This result is similar to some earlier works [2,8]. We also find that there is no significant relationship between the length of a bidirectional promoter and the degree of expression correlation (data not shown). This result is consistent with Trinklein's work [2].

### Functional similarity of bidirectional genes

In the previous studies about human genome, researchers noted that many bidirectional transcribed genes are related to DNA-repair mechanism [1,2]. It means that head-to-head structured genes may be associated functionally. To test this hypothesis, we analyzed the functional similarity of bidirectional genes using the Gene Ontology (GO) functional annotation (See Method for details). GO is a rooted directed acyclic graph (DAG) with three subcategories of "cellular component", "molecular function" and "biological process". Each GO node covers a set of genes with specific annotations and a gene is covered by one or multiple GO nodes along the DAG generally. The closer a node is to the terminal, the more detailed annotations are given. Similarly to Deng et al [19], those nodes that cover at least 50 genes and none of their offspring node satisfying this condition are defined as terminal informative nodes (TINs). We obtained 31 TINs in subcategory "cellular component", 82 TINs in "molecular function" and 125 TINs in "biological process" respectively, which can be found in the Additional file 1. Among all the 37019 genes of *Arabidopsis thaliana*, 17365 are covered by at least one TIN, namely 2898 genes with "cellular component" TINs, 11140 with "molecular function" TINs and 10242 with "biological process" TINs, respectively. We declared a pair is annotated by meaning that both genes of the pair are covered by at least one TIN in a specific subcategory and assigned functional similarity as 1 if the two genes have overlapping TINs and 0 otherwise. Among all the 327 annotated bidirectional gene pairs, we found 34 pairs (10.4%) of them having functional similarity (functional similarity is 1) and 293 pairs having no functional similarity (functional similarity is 0), whereas the counterpart number in the 10000 random pairs are 109 (6.21%) and 1645. Such a difference is significant with a p value 1.33 × 10^-3 ^if we use a binomial background distribution, which supports the hypothesis that bidirectional genes are more likely to be functionally associated than randomly paired genes.

### Coexpression levels on groups with different functional similarity

Using the 10000 random pairs' Pearson correlation coefficients as background distribution, we calculated p values of correlation coefficients of bidirectional gene pairs (See Methods for details). Lower p values mean more correlated in expression. We denoted pairs with functional similarities equal to 1 from the 2471 bidirectional gene pairs as group one and functional similarities equal to 0 as group two. Table 1 – Coexpression levels on different groups. gives the proportion of coexpressed pairs in different groups. Group one is significantly more correlated in expression (p value = 1.22 × 10^-5^) while group two is less significant (p value = 1.09 × 10^-2^) with a binomial distribution model. This result indicates that bidirectional gene pairs having functional similarity exhibit stronger expression correlations, which is consistent with [7].

**Table 1:** Coexpression levels on different groups.

	P < = 0.05	P > 0.05	Ratio
2471 bidirectional gene pairs	170	1222	12.21%

Fun_sim = 1	11	14	44%

Fun_sim = 0	31	142	17.92%

Ratio means the percentage of more correlated pairs in expression (Remark: pairs without available express data are excluded)

### Identification of transcriptional regulatory region (TRR)

In previous studies, putative bidirectional promoters are defined as intergenic regions between TSSs with not more than 1000 base pairs long. However, a region designated like that may not contain functional promoters since promoters can locate faraway from TSSs, and the two genes may locate "head-to-head" closely along genome by a chance. Beware of this and because of the difficulties for experimental verifications of promoters, we further scrutinize the DNA sequences between the bidirectional genes by HSL we recently developed to see if these sequences contain potential functional regulatory regions (See Methods for details).

**Figure 4:**
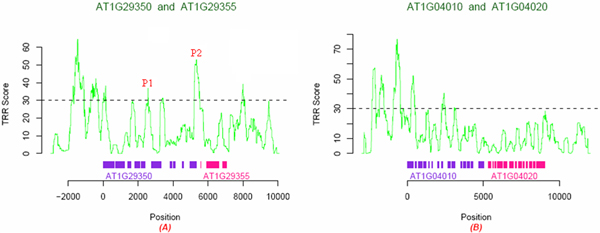
**TRR scores of genomic sequences**. The violet region represents a gene and the pink region represents another. Colored bars mean exons of gene, blank areas between the same colored bars are introns, and the only one blank region between the different colored bars in each figure is the generally defined bidirectional promoter. (A) There is only one peak P2 located in intergenic region, suggesting this region is a transcriptional regulatory region (TRR). There is also one peak P1 in this figure, but it is not located in intergenic region. (B) No peak in intergenic region, suggesting no TRR here.

We scanned the DNA sequences between bidirectional genes by HSL and assigned a TRR score for each position indicating the likelihood of the position-centered region being a transcriptional regulatory region (see Figure 4 – TRR scores of genomic sequences. for two examples). We chose threshold of TRR score 30 for *Arabidopsis thaliana *(For this threshold, the true positive rate is above 85% with false positive rate less than 20%. See paper submitted). We found that most (81.84%) of the intergenic regions between bidirectional genes with high TRR scores contain only one score peak, suggesting that the bidirectional genes share common promoter for transcription initiation.

### Functional similarity on high scoring TRR group and low scoring TRR group

Using the HSL program, we can divide all the 2471 bidirectional gene pairs into two groups, high scoring group (TRR score >= 30), and low scoring group (TRR score < 30). The percentage of pairs having functional similarity is 14.36% in the high scoring group, whereas 5.04% in the low TRR scoring group which is similar to that in random pairs (Table 2 – Functional similarity on different groups.). The high scoring group is more functionally associated than the two other groups dominantly. Simply modeling a binomial distribution on random pairs' function similarity, bidirectional gene pairs with TRR >= 30 are significantly correlated functionally with a p value 1.65 × 10^-5^, while low scoring group is not functionally associated at all (p value = 0.64). Bidirectional gene pairs have been confirmed more functionally associated by others [2,7]. However, bidirectional gene pairs with TRR < 30 are even worse than randomly paired genes (5.04% vs. 6.21%) in functional association and this implies us that bidirectional gene pairs in low scoring group are just close in position accidentally, without anything about biological correlation in co-regulation. Meanwhile, bidirectional gene pairs with TRR >= 30 are more dominantly associated functionally, suggesting they are more probably regulated divergently by a common promoter region through sharing essential regulatory elements working cooperatively.

**Table 2:** Functional similarity on different groups.

	Fun_sim = 1	Fun_sim = 0	Ratio
High scoring group	27	161	14.36%

Low scoring group	7	132	5.04%

Random pairs	109	1645	6.21%

Ratio means the percentage of pairs with higher functional similarity

### Predicting potential functional bidirectional promoters

Trinklein et al concluded that the majority of bidirectional promoters regulate the coexpression of a bidirectional gene pair [2], so gene pairs being more correlated in expression may be co-regulated by common bidirectional promoters. Table 1 – Coexpression levels on different groups. shows that bidirectional gene pairs having functional similarity exhibit stronger expression correlations, suggesting that functionally associated bidirectional gene pairs are probably co-regulated divergently by common bidirectional promoter. As described above, functionally associated bidirectional gene pairs with high TRR scores are probably regulated by common regulatory elements taking effects cooperatively. Therefore, we hypothesized that a functionally associated bidirectional gene pair with higher TRR score is probably co-regulated by a common promoter region through essential regulatory elements reacting on both genes cooperatively, and regarded the intergenic regions between them as potential functional bidirectional promoters. The correlation coefficient of expression was not taken into consideration due to the following reasons. Firstly, coexpression of a pair is not conserved under different experimental conditions. Secondly, the microarray data is often incomplete and we could not compute out all the correlation coefficients we want. According to the criteria of both having functional similarity and TRR score above 30, we predicted 27 bidirectional gene pairs to be regulated by common bidirectional promoters (Table 3 – True bidirectional promoters we predict).

**Table 3:** True bidirectional promoters we predict.

**ID**	**Pair1**	**Pair2**	**TRR**	**GO term**	**Function description**
1	AT1G17600	AT1G17610	34.0984	GO:0005524 MF GO:0004888 MF	ATP binding transmembrane receptor activity
2	AT3G51560	AT3G51570	34.6648	GO:0005524 MF GO:0004888 MF	ATP binding transmembrane receptor activity
3	AT1G72840	AT1G72850	34.9711	GO:0005524 MF GO:0004888 MF	ATP binding transmembrane receptor activity
4	AT4G36140	AT4G36150	47.5045	GO:0005524 MF GO:0004888 MF	ATP binding transmembrane receptor activity
5	AT5G40090	AT5G40100	52.8796	GO:0005524 MF GO:0004888 MF	ATP binding transmembrane receptor activity
6	AT2G23070	AT2G23080	31.2655	GO:0006468 BP	protein amino acid phosphorylation
7	AT1G07550	AT1G07560	38.9944	GO:0006468 BP	protein amino acid phosphorylation
8	AT5G35380	AT5G35390	40.0246	GO:0006468 BP	protein amino acid phosphorylation
9	AT3G46920	AT3G46930	44.4623	GO:0004712 MF GO:0006468 BP	protein serine/threonine/tyrosine kinase activity protein amino acid phosphorylation
10	AT3G57710	AT3G57720	49.5564	GO:0006468 BP	protein amino acid phosphorylation
11	AT3G01940	AT3G01950	36.6986	GO:0031225 CC	anchored to membrane
12	AT2G48130	AT2G48140	59.6507	GO:0008289 MF GO:0031225 CC GO:0006869 BP	lipid binding anchored to membrane lipid transport
13	AT5G37415	AT5G37420	32.2238	GO:0003700 MF	transcription factor activity
14	AT5G41020	AT5G41030	55.2906	GO:0003700 MF	transcription factor activity
15	AT4G38840	AT4G38850	35.204	GO:0009733 BP	response to auxin stimulus
16	AT5G18050	AT5G18060	36.3317	GO:0009733 BP	response to auxin stimulus
17	AT5G19420	AT5G19430	49.0875	GO:0008270 MF	zinc ion binding
18	AT2G28910	AT2G28920	54.0639	GO:0008270 MF	zinc ion binding
19	AT2G19720	AT2G19730	30.9849	GO:0003735 MF	structural constituent of ribosome
20	AT1G50940	AT1G50950	41.0419	GO:0009055 MF	electron carrier activity
21	AT4G30680	AT4G30690	44.2758	GO:0003743 MF	translation initiation factor activity
22	AT1G09080	AT1G09090	46.4263	GO:0009408 BP	response to heat
23	AT1G65130	AT1G65140	52.4771	GO:0016790 MF GO:0004843 MF	thiolester hydrolase activity ubiquitin-specific protease activity
24	AT5G47250	AT5G47260	52.5492	GO:0006511 BP	ubiquitin-dependent protein catabolic process
25	AT3G08000	AT3G08010	55.2916	GO:0003723 MF	RNA binding
26	AT5G01680	AT5G01690	56.6963	GO:0005451 MF	monovalent cation: proton antiporter activity
27	AT5G45250	AT5G45260	62.0555	GO:0042742 BP	defense response to bacterium

Pair1 and pair2 represent the loci of two genes in a bidirectional gene pair, and "CC", "MF" and "BP" are acronyms of "cellular component", "molecular function" and "biological process"

Among these 27 gene pairs, we found for each of five pairs (pair2, 4, 6, 7 and 26), the bidirectional genes contain the same functional protein domains and also share same orthologs from *Nematode*, *Yeast *and *Fungi*, which are evolutionally more ancient than *A. thaliana *(See Additional file 2 for details). The results indicate that genes in each of these five pairs are derived from a common ancestor into two genes during evolution.

Of the 27 bidirectional gene pairs, five pairs (pair 1–5 in Table 3 – True bidirectional promoters we predict.) sharing the same two GO terms GO:0005524 and GO:0004888, five pairs (pair 6–10) sharing the same GO term GO:0006468, two pairs (pair 11–12) sharing GO:0031225, two pairs (pair 13–14) sharing GO:0003700, two pairs (pair 15–16) sharing GO:0009733 and two pairs (pair 17–18) sharing GO:0008270. There are 20 GO terms being shared by identified bidirectional promoters in total, and most of these GO terms are supported by previous studies. Guarcuaglini et al found that murine RanBP1 and Htf9-c genes is regulated from a shared bidirectional promoter during cell cycle progression [20]. The Mouse Genome Informatics (MGI) database annotates Htf9-c with GO: 0003723 (RNA binding) which is listed in Table 3 – True bidirectional promoters we predict. [21]. Thymidine kinase (TK) is a growth factor inducible enzyme that is highly expressed in proliferating mammalian cells. Expression of mouse TK mRNA is controlled by transcriptional and posttranscriptional mechanisms including antisense transcription. Schuettengruber et al had identified a novel gene which is divergently transcribed from the bidirectional TK promoter, encoding kynenurine formamidase (KF), an enzyme of the tryptophan metabolism [5]. Murine gene TK is annotated in GO:0005524 (ATP binding) which is also in our prediction list by MGI annotation. Our prediction can also be supported by evidences in human genome. Xu et al concluded that the expression of human genes NBR2 and BRCA1 are co-ordinated through a bidirectional promoter [22], and Wu et al confirmed that BRCA1 have function GO:0008270 (zinc ion binding) which is shared by pair 17 and 18 [23]. Human genes PRKDC and MCM4 are divergently transcribed and located at chromosome 8 band q11 [24]. Gene PRKDC is annotated in GO:0018105 (peptidyl-serine phosphorylation) [25] which is a son node of GO:006468 (protein amino acid phosphorylation) in our prediction. Whereas MCM4 is covered by GO:0005524 (ATP binding) which is shared by pair 1–5 through the annotation of Gene Ontology. Besides that, Momota et al found that human genes COL4A3 and COL4A4 coding for the human alpha3(IV) and alpha4(IV) collagen chains are arranged head-to-head on chromosome 2q36 [26], while COL4A3 is annotated in GO:0006468 (protein amino acid phosphorylation) by Raya et al [27]. Bellizzi et al confirmed that two human genes SIRT3 and PSMD13 shared one bidirectional promoter [28], while gene PSMD13 is covered by GO:0031145 (anaphase-promoting complex-dependent proteasom-al ubiquitin-dependent protein catabolic process) through the annotation of Gene Ontology [29], which is a grandchildren of GO: 0006511 (ubiquitin-dependent protein catabolic process) we also predicted. Besides that, we find two genes from one bidirectional gene pair which is with lower TRR score not regulated by common bidirectional promoter. The two genes AT3G11450 and AT3G11460 constituting one bidirectional pair are located on chromosome 3 with a 773 base pairs long sequence separating them and the TRR score of this pair is 25.8, less than threshold 30. AT3G11450 is a heat shock gene [30], expression of which is regulated by heat stress transcriptional factors (HSFs), while AT3G11460 encodes pentatricopeptide repeat (PPR) protein. Geddy and Brown [31] revealed genes encoding PPR proteins are not conserved in location in *Arabidopsis *genomes and may be subject to diversifying selection. All these examples have been confirmed experimentally or by literature, providing a convincing support of our hypothesis. The five nodes GO:0006511, GO:0003723, GO:0008270, GO:000-6468 and GO:0005524 annotated most of genes mentioned above and also covered more than half of gene pairs we predicted in Table 3 – True bidirectional promoters we predict.

## Discussion

Bidirectional gene organization is reported existing in mammalian genomes by many literatures[1-3,32,33], but no genome-wide study of bidirectional genes for plant genome. We perform a systematic survey for the bidirectional genes in *Arabidopsis thaliana *and find this head-to-head gene structure is also very common, about 13.3% of the all the *A. thaliana *genes are arranged in such architectural features. Similarly to other studies [2,6], bidirectional genes are more correlated in expression and associated in function. Furthermore we find that bidirectional gene pairs sharing similar functions exhibit stronger expression correlations.

In previous studies, the intergenic region between two transcription start sites (TSSs) of a bidirectional gene pair is commonly designated as a putative bidirectional promoter. However, it is not clear whether this region has regulatory activity, or enriched of regulatory elements which are essential for initiation of transcription. We scanned the intergenic sequences by HSL and assigned a TRR score of it to make sure it contains transcriptional regulatory regions or not. It's interesting that most of the intergenic regions of bidirectional genes with high TRR scores contain only one score peak, suggesting that the bidirectional genes share common promoter for transcription initiation. Statistical results showed that functionally associated bidirectional gene pairs with high TRR scores are probably regulated by common regulatory elements taking effects cooperatively.

If we restrict ourselves to the high scored TRRs with functional associated bidirectional genes, we obtain 27 gene pairs, 5 pairs of which may be involved in the same biological process and share common orthlogs from *Nematode*, *Yeast *and *Fungi*. Surprisingly, almost all of them limited to several GO functions such as ATP binding et al., and most of them are supported by previous studies.

## Conclusion

Individual examples of divergently transcribed gene pairs have been found for years, most of which concern human genome [22,24,26,34] or mammalian genome [35,36], but corresponding results on plants are rarely reported. We conducted a systematic analysis of bidirectional gene pairs for *Arabidopsis thaliana *genome. We found that bidirectional gene pairs account for a large proportion (13.3%) of all the *A. thaliana *genes, confirming that this structure is also prevalent in plant genome. Similarly to other studies, we found that bidirectional gene pairs are more correlated in expression and associated functionally. Furthermore, we also perform systematic promoter activity analysis. We evaluated whether the putative bidirectional promoter regions are enriched of regulatory elements which are essential for promoter function and finally predicted 27 pairs of bidirectional genes which share common bidirectional promoters more probably. Five of them have the same functions for sharing common ancient orthologs, and in addition, more than half of them are further supported by sharing the same functional categories (GO terms) as these of handful experimental verified bidirectional genes in other organisms. These results significantly expanded the knowledge of "head-to-head" gene organization in plant genome and our work provides a new approach for further study of bidirectional genes in other genomes.

## Methods

### Identify bidirectional gene pairs and neighboring gene pairs

We obtained the information of annotated genes of *Arabidopsis thaliana *including loci, orientation of DNA chains and so on from TAIR [30]. There are 37019 genes in total and we defined the transcription start site coordinate and transcription end coordinate as 5' boundary and 3' boundary of a gene's locus. We then calculated the distances between 5' ends and 3' ends for genes in the same region no matter in forward chain or reverse. A gene pair that located in opposite strands and with the distance between their 5' ends not more than 1000 base pairs is defined as "bidirectional". We called a gene pair "convergent" if they located in opposite strands and with the distance between their 3' ends not more than 1000 bps, "codirectional" if they located in the same strand and with the distance between their 5' ends or 3' ends not more than 1000 bps. When studying coexpression, we refer the "convergent" genes and "codirectional" genes as neighboring gene pairs.

### Calculation of correlation coefficients using microarray data

The AtGenExpression project [37] includes many microarray data for comparing *Arabidopsis thaliana *responses to the biotic and abiotic environment. We downloaded the microarray data of 46 groups from its database and then complied the data from the same group to a data set. We normalized the data by quantile normalization before calculating the Pearson correlation coefficients for three kinds of gene pairs (bidirectional pairs, neighboring pairs and random pairs) in each of the 46 data sets independently.

### Download Gene Ontology data

We downloaded the three ontology files of subcategories "cellular component", "molecular function", "biological process" and the annotation file of *Arabidopsis thaliana *from Gene Ontology [38] on January 31, 2008. And using these files, we defined 31 terminal informative nodes (TINs) in subcategory "cellular component", 82 TINs in "molecular function" and 125 TINs in "biological process" respectively.

### Calculate p value of expression correlation coefficients

We denoted 10000 random pairs' Pearson correlation coefficients as background distribution B, and C_i _as the correlation coefficient of bidirectional gene pair i, computing p value P_i _through (1). Lower p values mean more correlated in expression.

(1) $$P_{\text{i}}=\frac{\#(B>C_{\text{i}})}{\text{\#}B}$$

### Brief description of HSL algorithm

We presented a hierarchical stochastic language (HSL) algorithm for the detection of transcriptional regulatory regions (TRRs) in eukaryotes [14]. Since in higher eukaryotes the promoter region of a gene is not easy to determine most of the time, whereas in yeast it usually coincides with the immediate upstream region of the gene, the upstream regions of the genes of yeast were used as putative TRR sample sequences (positive samples) for training of the algorithm. In the training procedures of HSL, words of length six are counted in yeast putative TRR regions as well as in coding/randomized regions. Then their occurrence frequencies in both cases are compared, where a high frequency in the first and a low one in the latter case can now be used as an indicator for a TRR region. This first-order analysis is extended in such a way that now the frequencies of word pairs are compared and constructed with likelihood ratio scores. (In principle this can be further extended to triplets, but we showed that the analysis with pairs seems to be sufficiently accurate.)

In the predication procedures, for a given DNA sequence, sum of scores of appeared word pairs was given for each window of L base pairs along the sequence to indicate the likelihood of core TRR, where L can be chosen based on the required resolution (we used L = 300 in this study). A threshold can be given to distinguish the TRRs from non-TRRs. We found that HSL model trained based on yeast achieved comparable accuracy in predicting TRRs in other species, e.g., fruit fly, human, and rice, thus demonstrated HSL model can be used to accurately predict core TRRs of transcripts across species

## Competing interests

The authors declare that they have no competing interests.

## Authors' contributions

LW and MPQ developed HSL program. MHD provided the method evaluating functional similarity by TIN. DYL and LHZ motivate the project. LHZ, MPQ and MHD propose the main idea. QW implemented the methods and analyzed the data. QW, LW and MHD wrote the manuscript.

## Supplementary Material

Additional file 1 A sketch of terminal informative nodes (TINs) and a specific list of TINs we obtained. Click here for file

Additional file 2 orthologs of pair2, 4, 6, 7 and 26 in several other species. Click here for file
