# Supplementary material for: Searching for bidirectional promoters in Arabidopsis thaliana
Source: BMC Bioinformatics. 2009 Jan 30;10(Suppl 1):S29. doi: 10.1186/1471-2105-10-S1-S29 (PMC2648788; doi:10.1186/1471-2105-10-S1-S29)
Supplement: Additional file 1 — A sketch of terminal informative nodes (TINs) and a specific list of TINs we obtained. [file 1471-2105-10-S1-S29-S1.pdf]

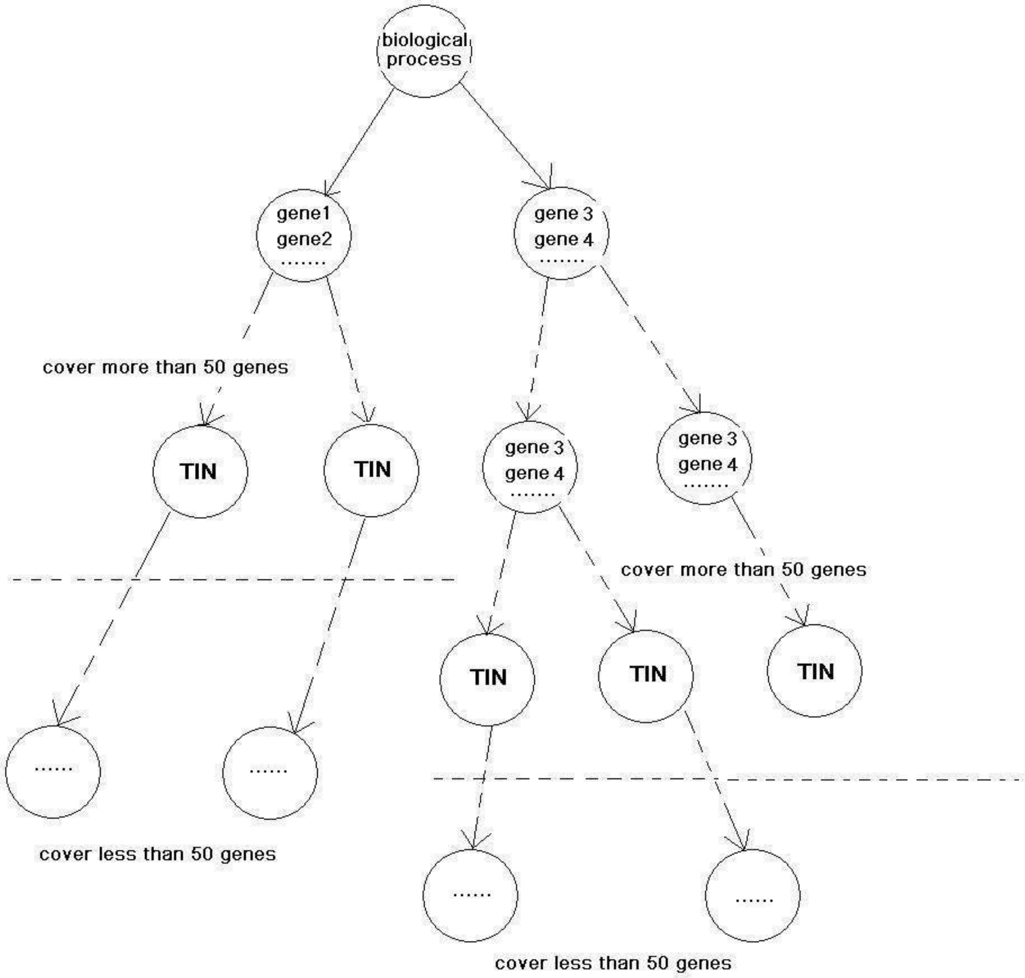

Sketch of TIN: A Directed Acyclic Graph (DAG) structured tree (A example of "biological process"). Each node may cover several or many genes, and Terminal Informative Nodes (TINs) locate in the bottom layer covering more than 50 genes.

31 TINs in cellular component	
**GO term**	**Function description**
GO:0009505	cellulose and pectin-containing cell wall
GO:0030120	vesicle coat
GO:0022627	cytosolic small ribosomal subunit
GO:0044432	endoplasmic reticulum part
GO:0044431	Golgi apparatus part
GO:0005777	peroxisome
GO:0005743	mitochondrial inner membrane
GO:0044455	mitochondrial membrane part
GO:0031969	chloroplast membrane
GO:0009570	chloroplast stroma
GO:0009543	chloroplast thylakoid lumen
GO:0009535	chloroplast thylakoid membrane
GO:0010287	plastoglobule
GO:0015934	large ribosomal subunit
GO:0000313	organellar ribosome
GO:0005774	vacuolar membrane
GO:0005635	nuclear envelope
GO:0005732	small nucleolar ribonucleoprotein complex
GO:0005667	transcription factor complex
GO:0009523	photosystem II
GO:0000786	nucleosome
GO:0005875	microtubule associated complex
GO:0031968	organelle outer membrane
GO:0000502	proteasome complex
GO:0000151	ubiquitin ligase complex
GO:0005834	heterotrimeric G-protein complex
GO:0031225	anchored to membrane
GO:0016021	integral to membrane
GO:0016469	proton-transporting two-sector ATPase complex
GO:0008287	protein serine/threonine phosphatase complex
GO:0048046	apoplast

82 TINs in molecular function	
**GO term**	**Function description**
GO:0004601	peroxidase activity
GO:0030246	carbohydrate binding
GO:0050662	coenzyme binding
GO:0005509	calcium ion binding
GO:0005507	copper ion binding
GO:0005506	iron ion binding
GO:0008270	zinc ion binding
GO:0008289	lipid binding
GO:0030533	triplet codon-amino acid adaptor activity
GO:0003700	transcription factor activity
GO:0003723	RNA binding
GO:0003743	translation initiation factor activity
GO:0005524	ATP binding
GO:0005525	GTP binding
GO:0019825	oxygen binding
GO:0005516	calmodulin binding
GO:0003779	actin binding
GO:0031072	heat shock protein binding
GO:0046983	protein dimerization activity
GO:0015450	P-P-bond-hydrolysis-driven protein transmembrane transporter activity
GO:0051082	unfolded protein binding
GO:0046906	tetrapyrrole binding
GO:0008026	ATP-dependent helicase activity
GO:0015662	ATPase activity\, coupled to transmembrane movement of ions\, phosphorylative mechanism
GO:0019829	cation-transporting ATPase activity
GO:0016811	hydrolase activity\, acting on carbon-nitrogen (but not peptide) bonds\, in linear amides
GO:0030599	pectinesterase activity
GO:0004620	phospholipase activity

To be continued on next page

GO term	Function description
GO:0004521	endoribonuclease activity
GO:0004527	exonuclease activity
GO:0008081	phosphoric diester hydrolase activity
GO:0003993	acid phosphatase activity
GO:0015071	protein phosphatase type 2C activity
GO:0016790	thiolester hydrolase activity
GO:0004650	polygalacturonase activity
GO:0004843	ubiquitin-specific protease activity
GO:0004194	pepsin A activity
GO:0004289	subtilase activity
GO:0004185	serine carboxypeptidase activity
GO:0008237	metallopeptidase activity
GO:0003755	peptidyl-prolyl cis-trans isomerase activity
GO:0004842	ubiquitin-protein ligase activity
GO:0004812	aminoacyl-tRNA ligase activity
GO:0016831	carboxy-lyase activity
GO:0016836	hydro-lyase activity
GO:0030508	thiol-disulfide exchange intermediate activity
GO:0009055	electron carrier activity
GO:0004497	monooxygenase activity
GO:0016616	oxidoreductase activity\, acting on the CH-OH group of donors\, NAD or NADP as acceptor
GO:0016651	oxidoreductase activity\, acting on NADH or NADPH
GO:0016706	oxidoreductase activity\, acting on paired donors\, with incorporation or reduction of molecular oxygen\, 2-oxoglutarate as one donor\, and incorporation of one atom each of oxygen into both donors
GO:0016667	oxidoreductase activity\, acting on sulfur group of donors
GO:0016620	oxidoreductase activity\, acting on the aldehyde or oxo group of donors\, NAD or NADP as acceptor
GO:0016627	oxidoreductase activity\, acting on the CH-CH group of donors
GO:0008080	N-acetyltransferase activity

To be continued on next page

GO term	Function description
GO:0004364	glutathione transferase activity
GO:0035251	UDP-glucosyltransferase activity
GO:0016763	transferase activity\, transferring pentosyl groups
GO:0008483	transaminase activity
GO:0008757	S-adenosylmethionine-dependent methyltransferase activity
GO:0004702	receptor signaling protein serine/threonine kinase activity
GO:0004712	protein serine/threonine/tyrosine kinase activity
GO:0003899	DNA-directed RNA polymerase activity
GO:0008047	enzyme activator activity
GO:0046910	pectinesterase inhibitor activity
GO:0030414	protease inhibitor activity
GO:0005083	small GTPase regulator activity
GO:0004888	transmembrane receptor activity
GO:0000156	two-component response regulator activity
GO:0003777	microtubule motor activity
GO:0045735	nutrient reservoir activity
GO:0003735	structural constituent of ribosome
GO:0016563	transcription activator activity
GO:0005351	sugar\:hydrogen ion symporter activity
GO:0015103	inorganic anion transmembrane transporter activity
GO:0005261	cation channel activity
GO:0015082	di-\, tri-valent inorganic cation transmembrane transporter activity
GO:0005451	monovalent cation\:proton antiporter activity
GO:0015294	solute\:cation symporter activity
GO:0005244	voltage-gated ion channel activity
GO:0046915	transition metal ion transmembrane transporter activity
GO:0015932	nucleobase\, nucleoside\, nucleotide and nucleic acid transmembrane transporter activity

125 TINs in biological process	
**GO term**	**Function description**
GO:0045934	negative regulation of nucleobase\, nucleoside\, nucleotide and nucleic acid metabolic process
GO:0051093	negative regulation of developmental process
GO:0048585	negative regulation of response to stimulus
GO:0048522	positive regulation of cellular process
GO:0051726	regulation of cell cycle
GO:0006355	regulation of transcription\, DNA-dependent
GO:0009966	regulation of signal transduction
GO:0009909	regulation of flower development
GO:0035195	miRNA-mediated gene silencing
GO:0051239	regulation of multicellular organismal process
GO:0031347	regulation of defense response
GO:0045454	cell redox homeostasis
GO:0030005	cellular di-\, tri-valent inorganic cation homeostasis
GO:0009825	multidimensional cell growth
GO:0009826	unidimensional cell growth
GO:0050790	regulation of catalytic activity
GO:0031668	cellular response to extracellular stimulus
GO:0007169	transmembrane receptor protein tyrosine kinase signaling pathway
GO:0009738	abscisic acid mediated signaling
GO:0009873	ethylene mediated signaling pathway
GO:0007264	small GTPase mediated signal transduction
GO:0007126	meiosis
GO:0012501	programmed cell death
GO:0000904	cellular morphogenesis during differentiation
GO:0051301	cell division
GO:0006334	nucleosome assembly
GO:0043623	cellular protein complex assembly
GO:0006413	translational initiation
GO:0007018	microtubule-based movement
GO:0048193	Golgi vesicle transport
GO:0006605	protein targeting

To be continued on next page

GO term	Function description
GO:0006839	mitochondrial transport
GO:0006913	nucleocytoplasmic transport
GO:0045045	secretory pathway
GO:0009832	cellulose and pectin-containing cell wall biogenesis
GO:0042545	cell wall modification
GO:0016044	membrane organization and biogenesis
GO:0016568	chromatin modification
GO:0030036	actin cytoskeleton organization and biogenesis
GO:0009658	chloroplast organization and biogenesis
GO:0000154	rRNA modification
GO:0010026	trichome differentiation
GO:0010053	root epidermal cell differentiation
GO:0006096	glycolysis
GO:0009073	aromatic amino acid family biosynthetic process
GO:0006418	tRNA aminoacylation for protein translation
GO:0009066	aspartate family amino acid metabolic process
GO:0000096	sulfur amino acid metabolic process
GO:0006576	biogenic amine metabolic process
GO:0009813	flavonoid biosynthetic process
GO:0009108	coenzyme biosynthetic process
GO:0006779	porphyrin biosynthetic process
GO:0042446	hormone biosynthetic process
GO:0015986	ATP synthesis coupled proton transport
GO:0006633	fatty acid biosynthetic process
GO:0046148	pigment biosynthetic process
GO:0006414	translational elongation
GO:0042364	water-soluble vitamin biosynthetic process
GO:0044242	cellular lipid catabolic process
GO:0006511	ubiquitin-dependent protein catabolic process
GO:0016114	terpenoid biosynthetic process
GO:0008654	phospholipid biosynthetic process
GO:0008202	steroid metabolic process
GO:0009250	glucan biosynthetic process

To be continued on next page

GO term	Function description
GO:0006486	protein amino acid glycosylation
GO:0006457	protein folding
GO:0006468	protein amino acid phosphorylation
GO:0016567	protein ubiquitination
GO:0006733	oxidoreduction coenzyme metabolic process
GO:0015994	chlorophyll metabolic process
GO:0006310	DNA recombination
GO:0006281	DNA repair
GO:0006261	DNA-dependent DNA replication
GO:0006397	mRNA processing
GO:0008380	RNA splicing
GO:0043414	biopolymer methylation
GO:0016311	dephosphorylation
GO:0033554	cellular response to stress
GO:0007568	aging
GO:0048438	floral whorl development
GO:0048437	floral organ development
GO:0048507	meristem development
GO:0009793	embryonic development ending in seed dormancy
GO:0009553	embryo sac development
GO:0009555	pollen development
GO:0003002	regionalization
GO:0009640	photomorphogenesis
GO:0017038	protein import
GO:0006865	amino acid transport
GO:0008643	carbohydrate transport
GO:0006855	multidrug transport
GO:0009926	auxin polar transport
GO:0006820	anion transport
GO:0006813	potassium ion transport
GO:0000041	transition metal ion transport
GO:0006869	lipid transport
GO:0006857	oligopeptide transport

To be continued on next page

GO term	Function description
GO:0009814	defense response\, incompatible interaction
GO:0006118	electron transport
GO:0015980	energy derivation by oxidation of organic compounds
GO:0019684	photosynthesis\, light reaction
GO:0005984	disaccharide metabolic process
GO:0009856	pollination
GO:0042742	defense response to bacterium
GO:0050832	defense response to fungus
GO:0009624	response to nematode
GO:0019953	sexual reproduction
GO:0009651	response to salt stress
GO:0009642	response to light intensity
GO:0009411	response to UV
GO:0009409	response to cold
GO:0009408	response to heat
GO:0009414	response to water deprivation
GO:0009733	response to auxin stimulus
GO:0009735	response to cytokinin stimulus
GO:0009739	response to gibberellin stimulus
GO:0010038	response to metal ion
GO:0009753	response to jasmonic acid stimulus
GO:0009743	response to carbohydrate stimulus
GO:0006979	response to oxidative stress
GO:0009751	response to salicylic acid stimulus
GO:0009636	response to toxin
GO:0042594	response to starvation
GO:0009611	response to wounding
GO:0007623	circadian rhythm
