# Supplementary material for: Searching for bidirectional promoters in Arabidopsis thaliana
Source: BMC Bioinformatics. 2009 Jan 30;10(Suppl 1):S29. doi: 10.1186/1471-2105-10-S1-S29 (PMC2648788; doi:10.1186/1471-2105-10-S1-S29)
Supplement: Additional file 2 — orthologs of pair2, 4, 6, 7 and 26 in several other species. [file 1471-2105-10-S1-S29-S2.pdf]

Pairs with common orthologs			
**Pair_ID**	**Gene pairs**	**Species**	**Orthologs**
**2**	**AT3G51560<br>AT3G51570**	***Caenorhabditis remanei***	**cr01.sctg700.wum.2.1 (WBGene00073322)<br>cr01.sctg84.wum.23.1 (WBGene00066813)<br>cr01.sctg84.wum.25.1 (WBGene00066815)<br>cr01.sctg84.wum.21.1 (WBGene00066811)<br>cr01.sctg2713.wum.1.1 (WBGene00075677)<br>cr01.sctg84.wum.27.1 (WBGene00066817)<br>cr01.sctg84.wum.31.1 (WBGene00066821)<br>cr01.sctg3429.wum.1.1 (WBGene00076040)<br>cr01.sctg3279.wum.1.1 (WBGene00075974)**
**4**	**AT4G36140<br>AT4G36150**	***Caenorhabditis remanei***	**cr01.sctg700.wum.2.1 (WBGene00073322)<br>cr01.sctg84.wum.23.1 (WBGene00066813)<br>cr01.sctg84.wum.25.1 (WBGene00066815)<br>cr01.sctg84.wum.21.1 (WBGene00066811)<br>cr01.sctg2713.wum.1.1 (WBGene00075677)<br>cr01.sctg84.wum.27.1 (WBGene00066817)<br>cr01.sctg84.wum.31.1 (WBGene00066821)<br>cr01.sctg3429.wum.1.1 (WBGene00076040)<br>cr01.sctg3279.wum.1.1 (WBGene00075974)**
**6**	**AT2G23070<br>AT2G23080**	***Caenorhabditis briggsae***	**BP:CBP03097(WBGene00033711)**
		Caenorhabditis elegans	**WBGene00002191**
		Caenorhabditis remanei	**Cre-kin-3 (WBGene00056231)**
		Candida glabrata	**XP_447476**
		Cryptococcus neoformans	**XP_571600**
		Debaryomyces hansenii	**XP_456923**
		Schizosaccharomyces pombe	**SPAC23C11.11**
		Yarrowia lipolytica	**XP_502914**
**7**	**AT1G07550<br>AT1G07560**	***Caenorhabditis briggsae***	**BP:CBP20987 (WBGene00024957)**
		Caenorhabditis remanei	**cr01.sctg1121.wum.3.1 (WBGene00074176)<br>cr01.sctg33.wum.115.1 (WBGene00062774)**
**26**	**AT5G01680<br>AT5G01690**	***Caenorhabditis remanei***	**cr01.sctg700.wum.2.1 (WBGene00073322)<br>cr01.sctg84.wum.23.1 (WBGene00066813)<br>cr01.sctg84.wum.25.1 (WBGene00066815)<br>cr01.sctg84.wum.21.1 (WBGene00066811)<br>cr01.sctg2713.wum.1.1 (WBGene00075677)<br>cr01.sctg84.wum.27.1 (WBGene00066817)<br>cr01.sctg84.wum.31.1 (WBGene00066821)<br>cr01.sctg3429.wum.1.1 (WBGene00076040)<br>cr01.sctg3279.wum.1.1 (WBGene00075974)**
